# Supplementary material for: SLITRK1-mediated noradrenergic projection suppression in the neonatal prefrontal cortex
Source: Commun Biol. 2022 Sep 9;5:935. doi: 10.1038/s42003-022-03891-y (PMC9463131; doi:10.1038/s42003-022-03891-y)
Supplement: Supplementary file 2 — Supplementary Information [file 42003_2022_3891_MOESM2_ESM.pdf]

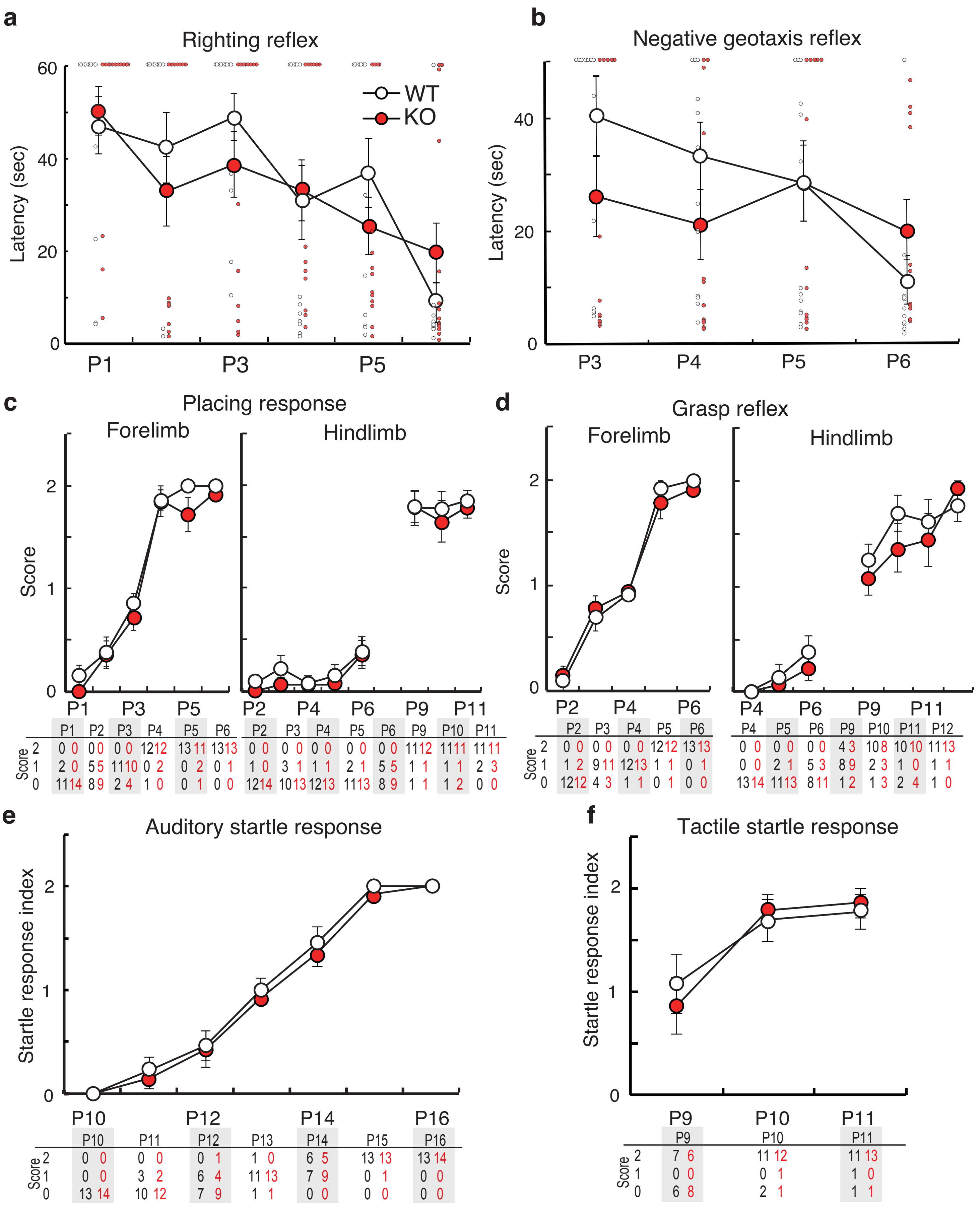

Supplementary Figure 1

Early postnatal behavior tests 1 (WT, *n* = 13; KO, *n* = 14). Results for (a) righting reflex, (b) negative geotaxis, (c) placing response, (d) grasp reflex, (e) auditory startle response, and (f) tactile startle response tests are indicated. Values are presented as means ± SEM. Tests were conducted as described in *Methods*. A click sound generator (O' HARA & Co.) was used for the auditory startle response test.

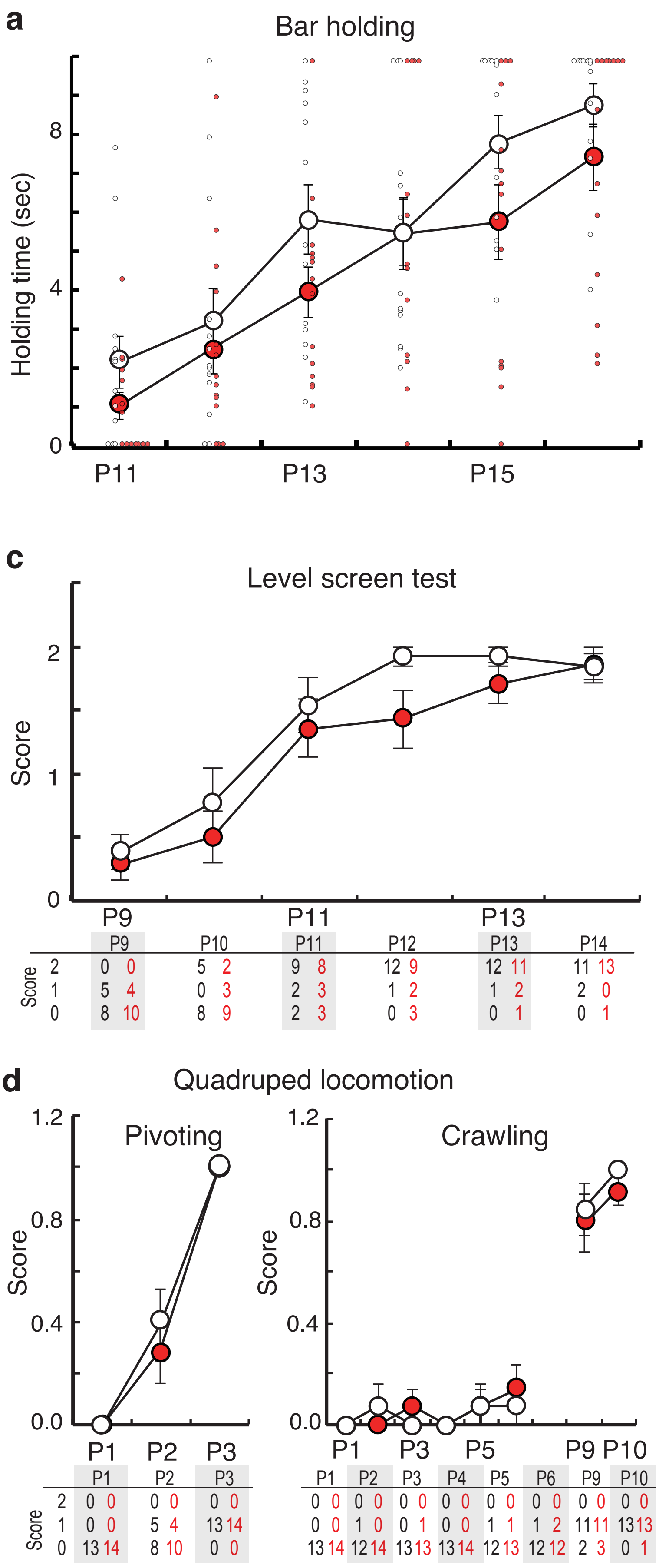

Supplementary Figure 2
Early postnatal behavior tests 2 (WT, $n = 13$; KO, $n = 14$). Results for (a) bar holding, (b) vertical screen, (c) level screen, and (d) quadruped locomotion. Values are presented as means ± SEM. Tests were conducted as described by *Methods*. *, $P < 0.05$ in *t*-test.

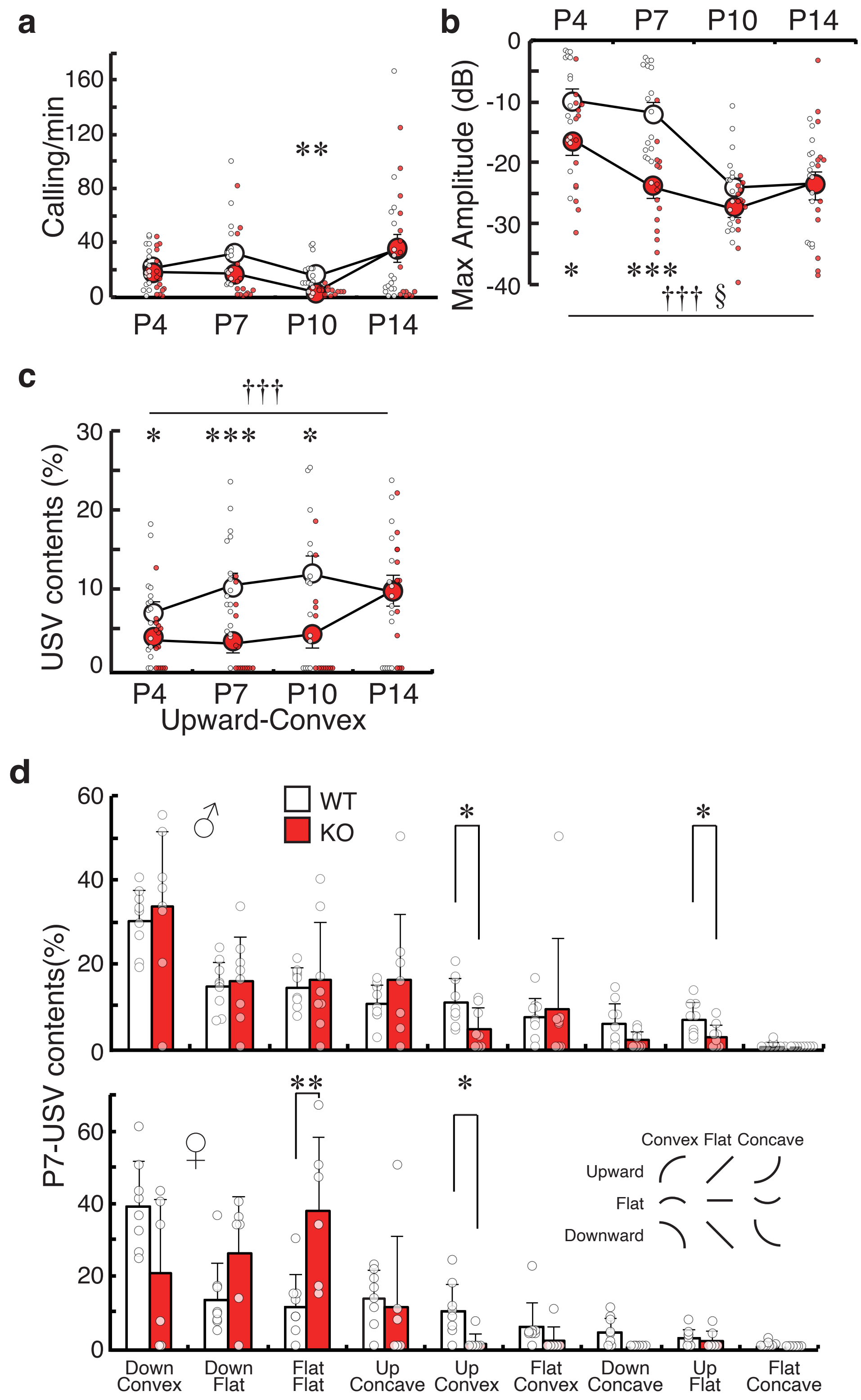

Supplementary Figure 3

Ultrasonic vocalization of isolated pups (supplementary results for Figure 1F). Calling rate (a), maximum amplitude (b), and USV contents (c) for mixed-sex. *n* = 17 (WT) and *n* = 14 mice (KO). (d) Voice elements were categorized into nine tone profiles based on the downward-flat upward and convex-flat-concave criteria as illustrated. The frequency of each profile was presented as a percentage. Vocalizations from Slitrk1 WT and KO mice at P7 underwent sex-based analysis. WT male, *n* = 9; KO male, *n* = 8; WT female, *n* = 8; KO female, *n* = 6. Values are presented as mean ± SD. *, $P < 0.05$; ** $P < 0.01$; ***, $P<0.001$ in *t*-test. †††, $P < 0.001$ in two-way ANOVA (genotype and day as main factors, genotype effect). §, $P < 0.05$ in two-way ANOVA (genotype × day interaction).

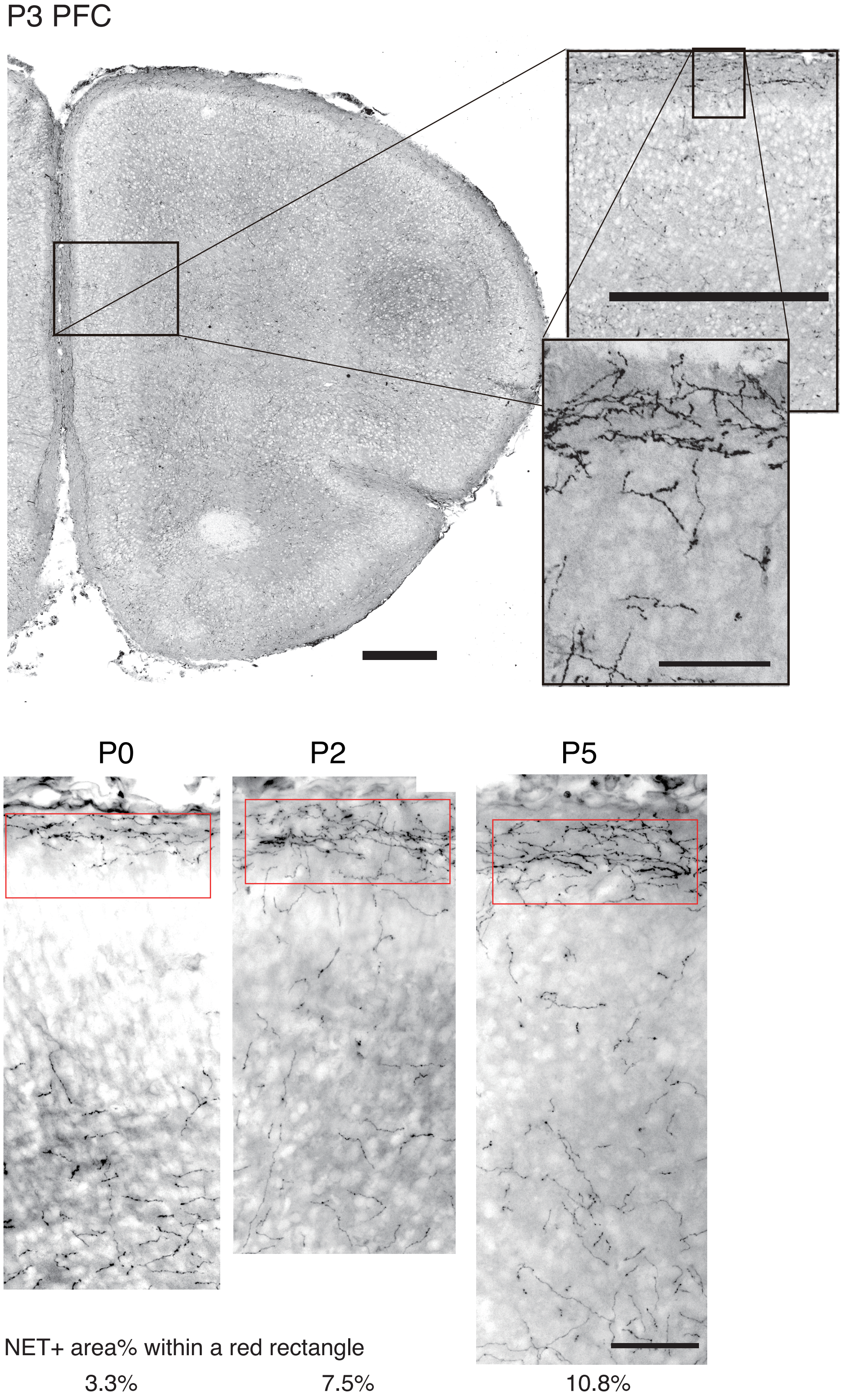

Supplementary Figure 4
Normal noradrenergic fiber development in the PFC of mouse neonates. The coronal sections through the PFC obtained from WT mice at P0, P2, and P5 were immunostained using an anti-NET antibody. NET positive area proportions in the red rectangle were indicated under each panel. Scale bar, 500 μm (thick); 50 μm (thin).

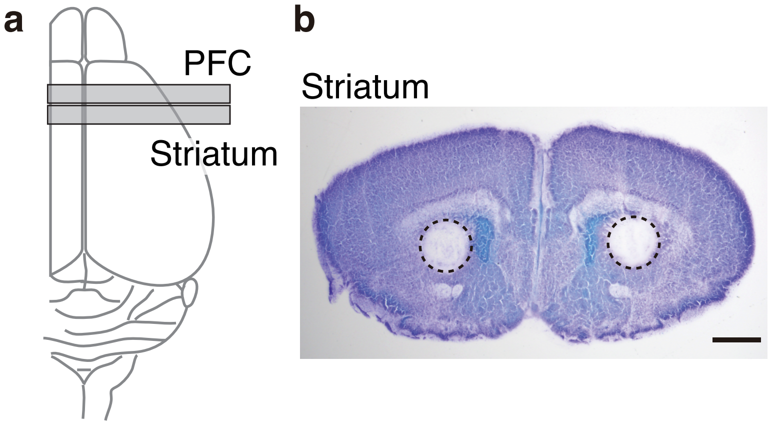

c

Monoamine contents (pmol/mg protein)

PFC	WT Male	WT Female	KO Male	KO Female
5HT	1.40±0.3	2.26±0.7	1.50±0.3	1.86±0.4
DA	3.33±0.7	3.42±0.7	2.98±0.4	2.68±0.6
DOPAC	3.59±0.6	4.00±0.8	2.77±0.2	3.29±0.4
HIAA	0.51±0.2	1.24±0.4	0.57±0.1	0.95±0.4
HVA	2.67±0.5	4.43±1.2	4.68±0.5 *	4.20±1.1
MHPG †	81.83±33	76.31±28.9	24.80±9.8	29.49±9.7
NA †	2.58±0.6	4.70±0.9 ¶	4.87±0.4 **	5.25±0.6
HIAA/5HT	0.38±0.1	0.55±0.2	0.40±0.0	0.32±0.1
DOPAC/DA	1.24±0.2	1.20±0.1	1.10±0.1	1.43±0.4
MHPG/NA	35.71±16.9	16.33±5.9	4.95±1.7	7.02±2.7

†P<0.05 in ANOVA (Genotype), ¶P<0.05 in ANOVA (Sex), *P<0.05 in *t*-test

Striatum	WT Male	WT Female	KO Male	KO Female
5HT	1.60±0.1	2.73±0.4 ¶	2.27±0.4	2.92±0.3
DA	64.00±6.4	65.36±5.0	77.03±13.8	90.55±15.2
DOPAC	25.66±2.7	28.21±3.5	30.25±4.6	34.30±5.3
HIAA	0.60±0.1	1.61±0.5 ¶	0.71±0.3	1.34±0.3
HVA	8.06±0.9	11.46±1.4	10.05±2.1	11.22±1.8
MHPG	41.77±9.3	61.14±17.3	64.83±18.8	47.04±6.7
NA	0.69±0.2	0.67±0.2	1.02±0.2	0.87±0.1
HIAA/5HT	0.37±0.0	0.53±0.1 ¶	0.27±0.1	0.47±0.1
DOPAC/DA	0.42±0.1	0.39±0.0	0.41±0.0	0.37±0.0
MHPG/NA	125.69±60.0	165.02±67.1	146.37±79.3	52.25±13.8

¶P<0.05 in ANOVA (Sex)

Supplementary Figure 5

HPLC-based monoamine quantification. (a) Coronal brain sections were prepared from the illustrated areas. (b) Cresyl violet staining of the sections used for sampling. Positions of the punch biopsy are indicated by circle. Scale bar, 1mm. (c) Table for the absolute monoamine levels in the PFC and stratum of Slitrk1 WT and KO mice. The table presents data corresponding to those shown in Figure 4. n = 5 or 7 mice per genotype. Values are presented as mean ± SD. *, $P < 0.05$; **, $P < 0.01$ in *t*-test. †, $P < 0.05$ (genotype); ¶, $P < 0.05$ (sex) in two-way ANOVA (genotype and sex as main factors).

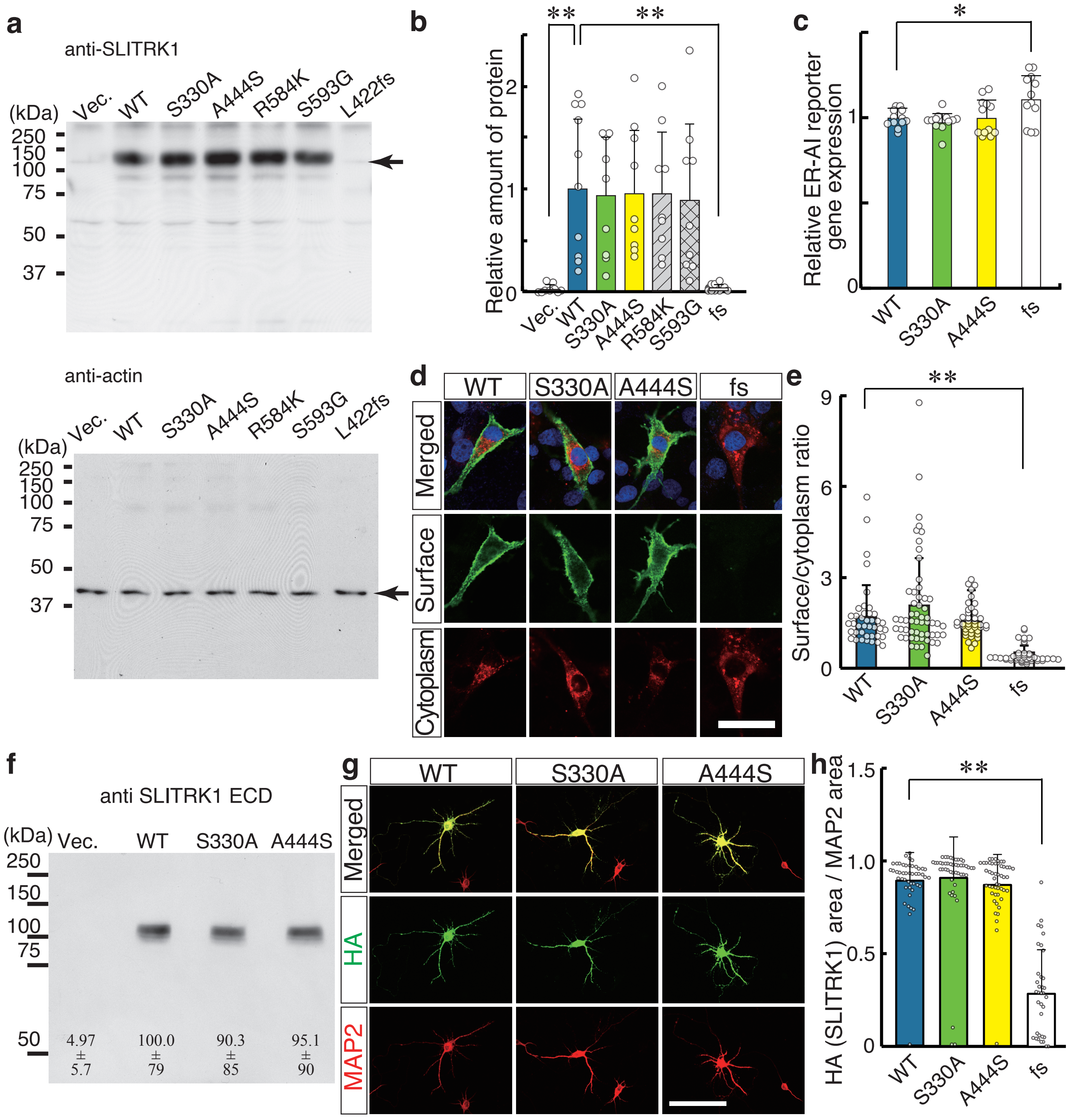

Supplementary Figure 6

The basic properties of SLITRK1 mutants. (a, b) Expression levels in transfected PC12 cells were quantified through immunoblotting analysis (a, $n = 9$ experiments). (b) There were no significant differences among WT, S330A, and A444S. (c) ER stress was measured for COS7 cells through co-transfection with ER stress-responsive Luciferase reporter pERAI-Luc ($n = 12$ wells from 4 experiments). (d, e) The subcellular distribution of SLITRK1 mutants in COS7 cells (WT, $n = 40$; S330A, $n = 55$; A444S, $n = 42$; fs, $n = 45$ cells). (d) A representative cell image. (e) The ratio of cell surface/cytoplasmic signals was comparable among WT, S330A, and A444S. (f) The secreted SLITRK1 ECD from COS7 transfectants was quantified through immunoblotting analysis using anti-SLITRK1 N-terminus antibody (R&D Systems). Densitometric quantification results are indicated at the bottom (Relative protein levels where WT = 100%. Mean ± SD from $n = 10$ experiments). (g, h) Mutant protein distributions in primary cultured hippocampal neurons. (g) Neurons transfected with HA-tagged SLITRK1 or its variants were stained using anti HA (green) and MAP2 (red). (h) The ratio of HA^{+} area to $MAP2^{+}$ area was comparable among WT, S330A, and A444S (WT, $n = 46$; S330A, $n = 47$; A444S, $n = 49$; fs, $n = 35$ cells). Scale bar, 50 μm (d) and 100 μm (g). Values are presented as mean ± SD. *, $P < 0.05$; **, $P < 0.01$ in *Dunnett' s* test, compared with WT.

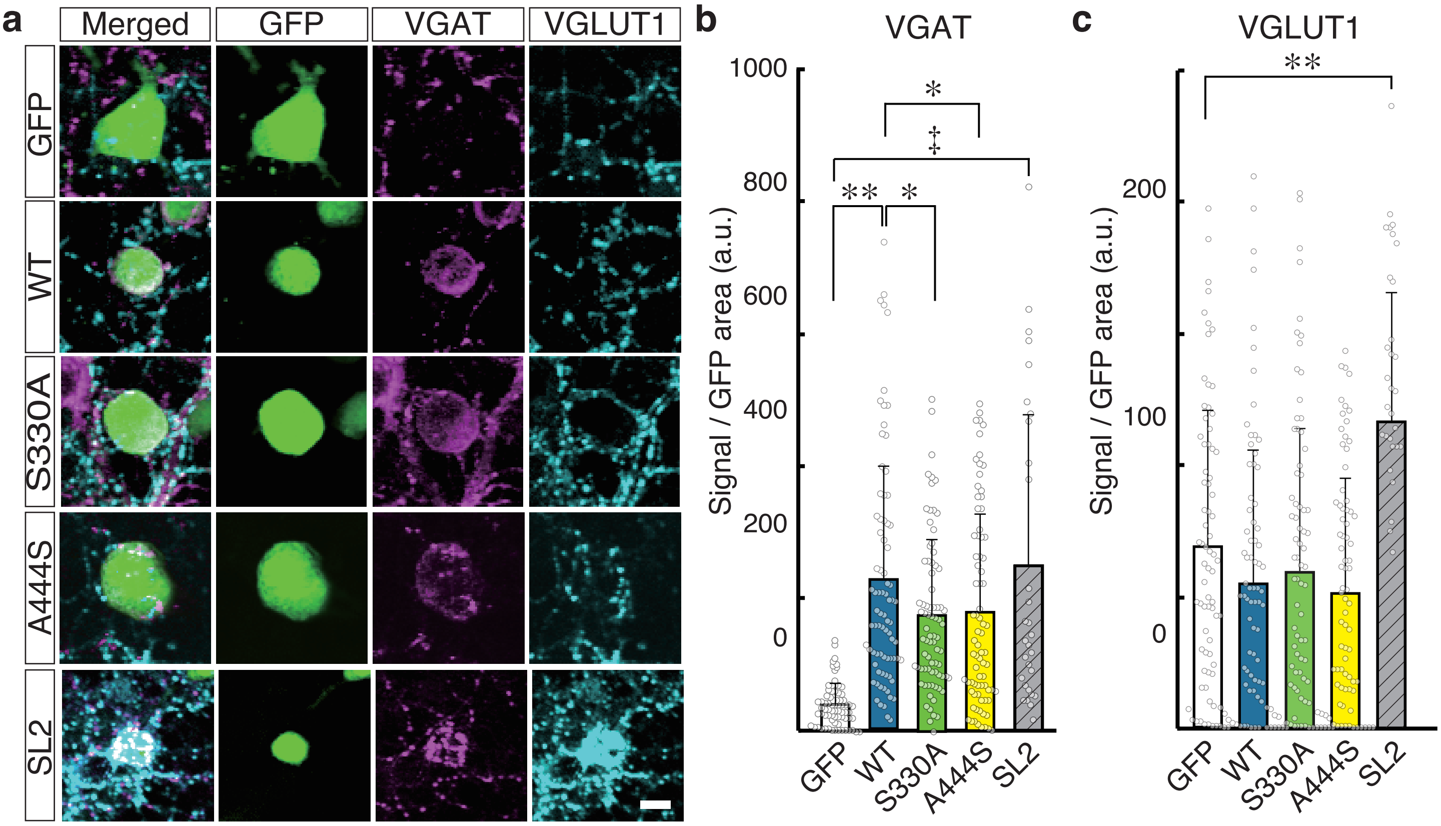

Supplementary Figure 7

The synapse-inducing activity was altered in SLITRK1 mutants. HEK293T transfectants expressing SLITRK1 or its mutants were co-cultured with hippocampal neurons. Two days after co-culture, induced pre-synapses were quantified through VGAT or VGLUT1 immunostaining (GFP, *n* = 74; WT, *n* = 80; S330A, *n* = 79; A444S, *n* = 79; SL2, *n* = 30). (a) Representative images of the synaptogenic assay. (b, c) Quantitative analysis of the VGAT (magenta, b) and VGLUT1 (cyan, c). Transfected 293T cells were identified by GFP signal (green). SL2, SLITRK2; Scale bar, 10 μm. Values are presented as mean ± SD. *, $P < 0.05$; **, $P < 0.01$ in Dunnett' s test, compared with WT transfectants in (b). ‡, $P < 0.05$ in *t*-test between GFP and WT transfectants. In (c), **, $P < 0.01$ in *Dunnett' s* test, compared with GFP.

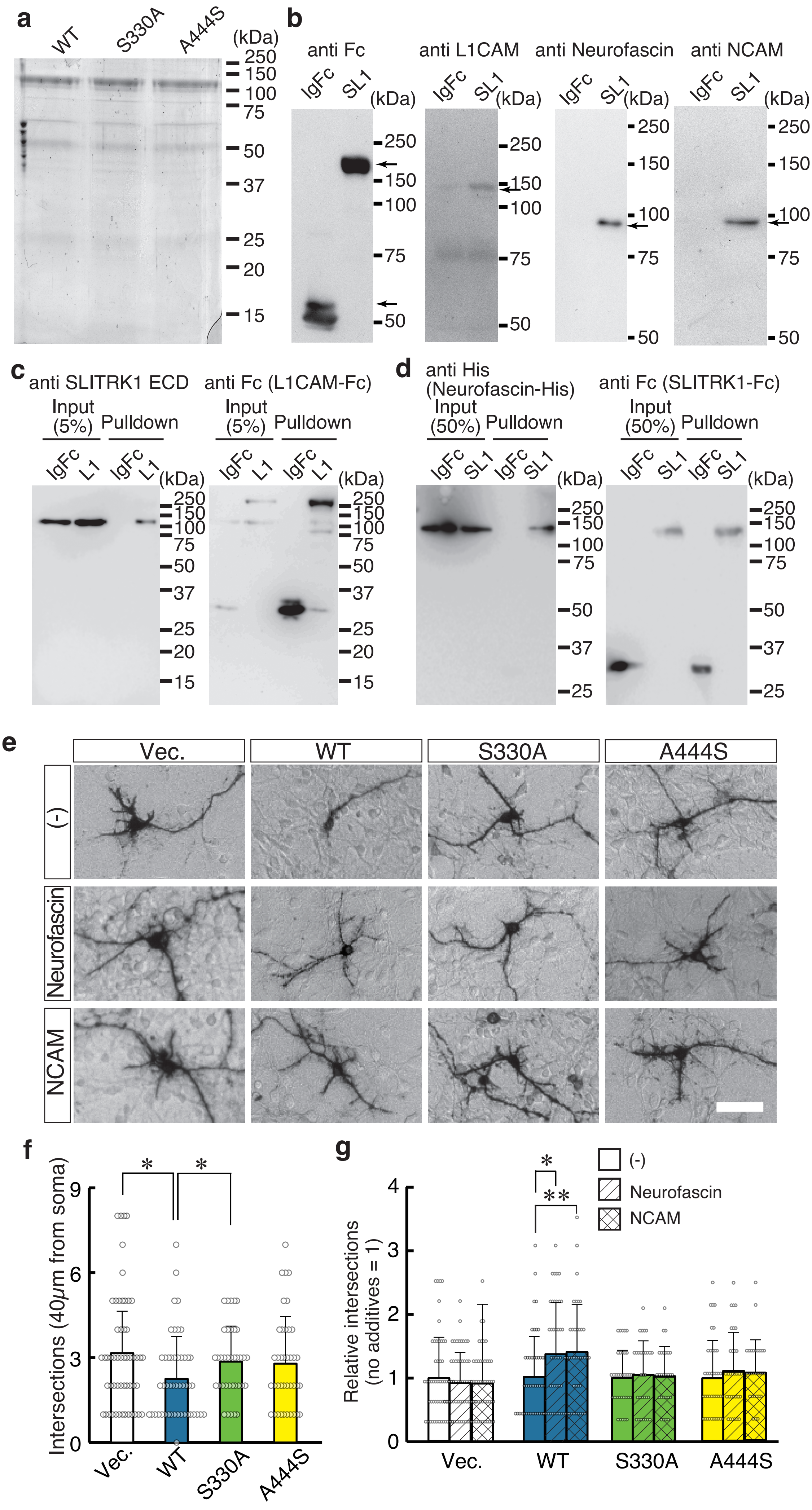

a
WT
S330A
A444S
(kDa)
250
150
100
75
50
37
25
20
15
b
anti Fc
anti L1CAM
anti Neurofascin
anti NCAM
IgFc
SL1
c
anti SLITRK1 ECD
anti Fc (L1CAM-Fc)
Input (5%)
Pulldown
L1
d
anti His (Neurofascin-His)
anti Fc (SLITRK1-Fc)
Input (50%)
e
Vec.
WT
S330A
A444S
(-)
Neurofascin
NCAM
f
Intersections (40μm from soma)
g
Relative intersections (no additives = 1)
(-)
Neurofascin
NCAM

Supplementary Figure 8

Supplementary results regarding the SLITRK1-L1 family interaction. (a) Recombinant SLITRK1 ECD-Fc fusion proteins were used for the pull-down assay. A silver-stained SDS-PAGE gel image. A major band between 100 and 150 kDa markers corresponds to the recombinant proteins. (b) A pulldown assay using SLITRK1 ECD-Fc (bait) and brain lysate. Coprecipitants were detected with specific antibody. The arrows indicate the major bands detected by the antibodies indicated above. (c, d) Pulldown assays showing the interaction between recombinant SLITRK1 ECD and L1CAM-Fc (c) and Neurofascin-His and SLITRK1 ECD-Fc (d). The antibodies used for immunoblotting are indicated above. All blot images are uncropped. (e-g) SLITRK1-L1 family interactions increase neurite complexity. Primary culture of the somatosensory cortex after *in utero* electroporation of SLITRK1 or its mutants and treated with Neurofascin (8.5 μg/mL in medium) or NCAM protein (8.5 μg/mL in medium) (Vec.+(-), $n = 54$; Vec.+Neurofascin, $n = 55$; Vec.+NCAM, $n = 49$; WT+(-), $n = 48$; WT+Neurofascin, $n = 41$; WT+NCAM, $n = 38$; S330A+(-), $n = 36$; S330A+Neurofascin, $n = 30$; S330A+NCAM, $n = 27$; A444S+(-), $n = 39$; WT+Neurofascin, $n = 32$; WT+NCAM, $n = 30$ cells). (e) Representative cell images. The transfected neurons were identified by detecting the alkaline phosphatase activity derived from backbone vector. Scale bar, 50 μm. (f) The number of intersections between neurites and circles with a 40 μm radius, centered at the soma, in the absence of L1 family proteins. (g) Intersections of Neurofascin- or NCAM-treated cells. For each transfectant, the intersection number without L1 family proteins ([-], open box) was defined as 100%. Values are presented as mean ± SD. *, $P < 0.05$; **, $P < 0.01$ in *U*-test (f) or *Steel’ s* test (g).

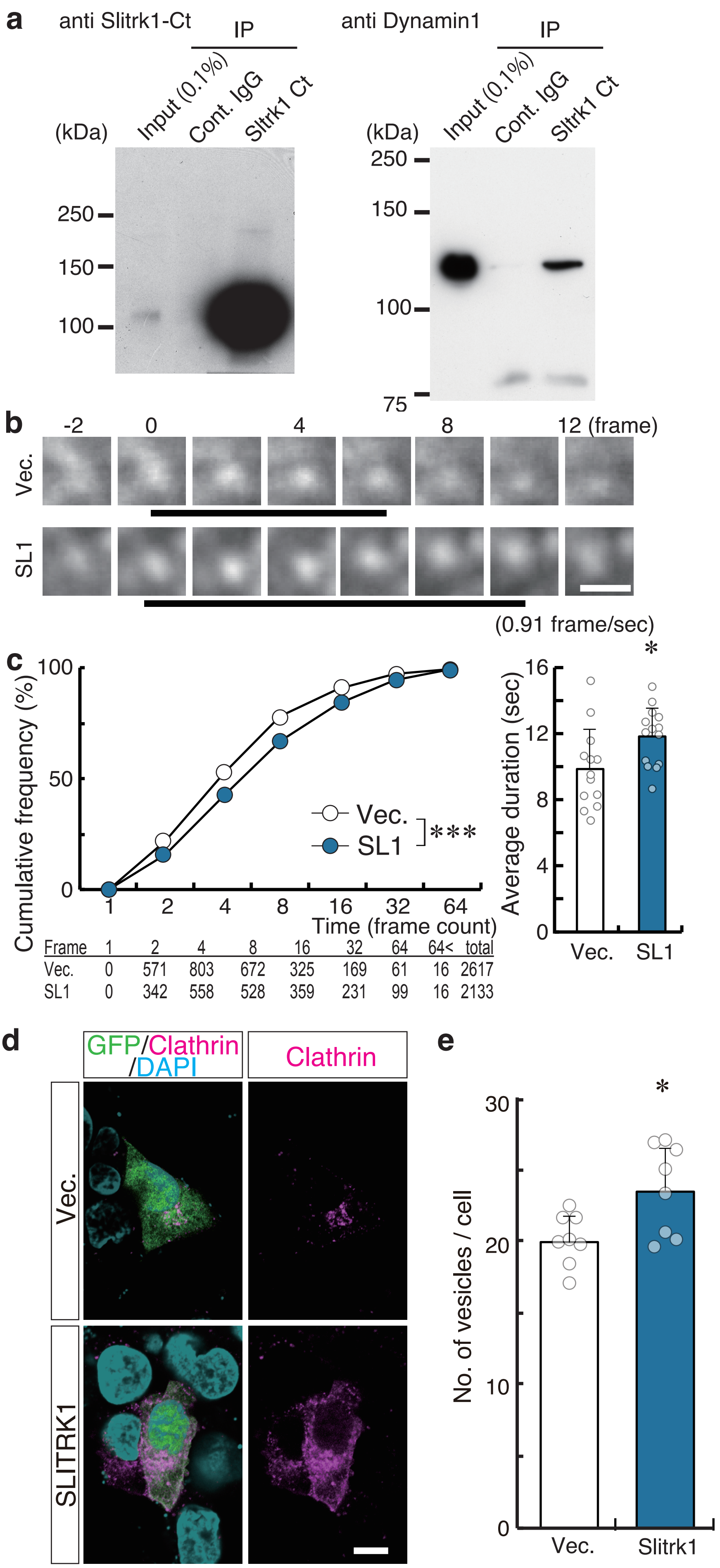

a
anti Slitrk1-Ct
IP
Input (0.1%)
Cont. IgG
Sltrk1 Ct
(kDa)
250
150
100
anti Dynamin1
IP
Input (0.1%)
Cont. IgG
Sltrk1 Ct
(kDa)
250
150
100
75
b
-2
0
4
8
12 (frame)
Vec.
SL1
(0.91 frame/sec)
c
Cumulative frequency (%)
100
50
0
1
2
4
8
16
32
64
Time (frame count)
Vec.
SL1
***
Frame 1 2 4 8 16 32 64 64< total
Vec. 0 571 803 672 325 169 61 16 2617
SL1 0 342 558 528 359 231 99 16 2133
Average duration (sec)
*
16
12
8
4
0
Vec.
SL1
d
GFP/Clathrin/DAPI
Clathrin
Vec.
SLITRK1
e
No. of vesicles / cell
*
30
20
10
0
Vec.
Slitrk1

Supplementary Figure 9

Slitrk1 can interact with Dynamin1 and extend the duration of clathrin vesicles. (a) Immunoprecipitation using anti-Slitrk1 antibody and mouse brain lysates. Precipitates underwent immunoblotting analysis using anti-Slitrk1 or anti-Dynamin1 antibody. (b) Time-lapse imaging for NGF-induced endocytosis in PC12D cells expressing mCherry-tagged Clathrin with (SL1) or without (Vec.) SLITRK1. *Top numerals* indicate the frame number where the initial frame for vesicular detection is defined as zero. *Underlines* indicate the vesicle duration. (c) The distribution of the durations is demonstrated as cumulative frequency curves (Vec, n = 2617; SL1, n = 2133 vesicles for each condition); further, the average duration in each cell is shown in the bar graph (n = 13 or 14 cells for each condition). (d) Representative images of the analyzed PC12 cells. Scale bar, 10 μm. (e) The total number of Clathrin-mCherry^{+} vesicles in a cell. n = 8 experiments. Values in bar graphs are presented as mean ± SD. *, $P < 0.05$ in the t-test. ***, $P < 0.001$ in *Kolmogorov–Smirnov* test.

mRNA levels in PFC at P3 (WT=1)

	WT		KO		*P*-value		
Gene	Male	Female	Male	Female	Male	Female	Both sexes
Sema3a	1.00±0.12	1.00±0.16	0.90±0.090	0.86±0.06	0.08	0.11	0.01*
Sema3c	1.00±0.13	1.00±0.23	0.99±0.33	0.89±0.26	0.98	0.48	0.28
Sema3f	1.00±0.22	1.00±0.23	0.84±0.27	0.98±0.11	0.35	0.90	0.45
Sema7a	1.00±0.16	1.00±0.16	0.91±0.21	1.02±0.29	0.34	0.92	0.54
EphrinA5	1.00±0.37	1.00±0.20	0.82±0.33	1.01±0.22	0.44	0.94	0.66
EphrinB2	1.00±0.33	1.00±0.24	1.12±0.15	0.97±0.11	0.46	0.79	0.68
Slit1	1.00±0.16	1.00±0.17	0.93±0.06	0.91±0.14	0.24	0.41	0.13
Slit2	1.00±0.11	1.00±0.26	0.85±0.16	0.84±0.08	0.05*	0.25	0.02*
Slit3	1.00±0.13	1.00±0.18	0.92±0.17	0.76±0.11	0.31	0.03*	0.02*
BMP2	1.00±0.33	1.00±0.21	0.97±0.45	0.91±0.16	0.92	0.47	0.39
Netrin	1.00±0.26	1.00±0.23	1.20±0.61	0.98±0.19	0.55	0.90	0.54
Wnt5a	1.00±0.18	1.00±0.12	1.13±0.25	1.14±0.22	0.26	0.26	0.10
Wnt7b	1.00±0.16	1.00±0.11	1.03±0.41	1.05±0.22	0.88	0.67	0.41
Shh	1.00±0.59	1.00±0.33	0.76±0.26	1.00±0.26	0.39	0.98	0.56
Ntf3	1.00±0.33	1.00±0.11	1.08±0.32	1.07±0.13	0.70	0.41	0.15
BDNF	1.00±0.45	1.00±0.19	0.83±0.17	0.93±0.23	0.42	0.63	0.21
GDNF	1.00±0.49	1.00±0.29	1.22±0.90	1.19±0.90	0.68	0.68	0.29
MAO A	1.00±0.21	1.00±0.13	0.93±0.28	1.04±0.1	0.70	0.56	0.97
MAO B	1.00±0.03	1.00±0.15	1.02±0.03	1.00±0.11	0.37	0.97	0.97
COMT	1.00±0.07	1.00±0.15	0.99±0.08	1.08±0.05	0.86	0.29	0.46
α2a	1.00±0.24	1.00±0.12	0.90±0.18	0.94±0.12	0.36	0.42	0.23
VMAT2	1.00±0.27	1.00±0.17	0.81±0.25	0.92±0.21	0.17	0.52	0.12
Slitrk1	1.00±0.36	1.00±0.25	0.00±0.00	0.00±0.00	0.00***	0.00***	0.00***

Consideration of multiple tests, *P*-values were verified with Benjamini-Hochberg procedure.

mRNA levels in PFC at P7 (WT=1)

	WT		KO		*P*-value		
Gene	Male	Female	Male	Female	Male	Female	Both sexes
Sema3a	1.00±0.23	1.00±0.59	1.30±0.93	0.82±0.54	0.48	0.60	0.82
Sema3c	1.00±0.31	1.00±0.23	1.71±1.98	0.74±0.28	0.42	0.10	0.61
Sema3f	1.00±0.45	1.00±0.34	0.95±1.25	0.84±0.46	0.93	0.51	0.72
Sema7a	1.00±0.19	1.00±0.25	1.68±2.02	0.66±0.31	0.45	0.06	0.71
EphrinA5	1.00±0.41	1.00±0.20	0.56±0.31	1.11±0.56	0.08	0.65	0.44
EphrinB2	1.00±0.31	1.00±0.21	1.31±1.26	0.59±0.39	0.57	0.05*	0.88
Slit1	1.00±0.10	1.00±0.35	1.14±0.21	0.93±0.12	0.34	0.75	0.78
Slit2	1.00±0.13	1.00±0.12	1.26±1.12	0.88±0.25	0.60	0.30	0.78
Slit3	1.00±0.29	1.00±0.25	1.04±0.90	0.83±0.16	0.93	0.19	0.73
BMP2	1.00±0.25	1.00±0.20	0.82±0.30	0.79±0.66	0.48	0.62	0.36
Wnt5a	1.00±0.30	1.00±0.44	1.42±1.45	0.39±0.30	0.51	0.02*	0.79
Wnt7b	1.00±0.29	1.00±0.51	1.96±1.87	2.15±1.96	0.27	0.22	0.07
Shh	1.00±0.39	1.00±0.17	1.89±1.94	0.94±0.50	0.32	0.80	0.34
Ntf3	1.00±0.91	1.00±0.33	0.90±0.25	0.70±0.31	0.84	0.14	0.38
BDNF	1.00±0.10	1.00±0.05	1.01±0.05	1.40±0.54	0.91	0.33	0.278
Slitrk1	1.00±0.07	1.00±0.30	0.05±0.02	0.09±0.04	0.00***	0.00***	0.00***

Consideration of multiple tests, *P*-values were verified with Benjamini-Hochberg procedure.

Supplementary Table 1
The gene expression profile of PFC tissue obtained from Slitrk1-KO mice at P3 and P7. Gene symbol, genotype, sex, and *P*-value are listed. The mean values for WT mice were adjusted to 1. Values are presented as mean ± SD. *, $P < 0.05$; ***, $P < 0.001$ in *t*-test. WT male $n = 4$, KO male $n = 6$ (MAO A, MAO B, Sema3c, Sema3f, EphA5, EphB2, BMP2, Netrin, Wnt7b, Shh, NTF3, BDNF, GDNF), WT male $n = 8$, KO male $n = 8$ (Sema3a, Sema7a, Slit1, Slit2, Slit3, Wnt5a, Slitrk1), WT and KO female $n = 5$.

Polymorphism	ID	Position	GRCh38 chr 13
p.Ser330Ala	rs145628951	c.988T>G	g.83880520A>C
p.Asp348Tyr	rs1232822038	c.1042G>T	g.83880466C>A
p.Gly352Arg	rs749499107	c.1054G>C	g.83880454C>G
p.Ala444Ser	rs1450785142	c.1330G>T	g.83880178C>A

Supplementary Table 2
List of NCBI dbSNP ID and position.

Excised band size	Accession	Gene	Symbol	MW [kDa]	Coverage [%]	#Peptides	Score	Localization*
200 kDa	Q810U3	Neurofascin	Nfasc	137.9	21	21	700	Plasma membrane
	P11627	Neural cell adhesion molecule L1	L1cam	140.9	5	4	188	Plasma membrane
	P13595	Neural cell adhesion molecule 1	Ncam1	119.4	2	2	113	Plasma membrane
	Q99104	Unconventional myosin-Va	Myo5a	215.4	1	2	82	Cytosol
100 kDa	Q60597	2-oxoglutarate dehydrogenase	Ogdh	116.4	32	25	889	Mitochondrial membrane
	P39053	Dynamin-1	Dnm1	97.7	32	24	783	Cytosol
	O88935	Synapsin-1	Syn1	74.1	8	4	155	Golgi apparatus
	Q78PY7	Staphylococcal nuclease domain -containing protein 1	Snd1	102	6	4	146	Cytosol
	O55029	Coatomer subunit beta'	Copb2	102.4	1	1	66	Golgi apparatus
	Q810C1	SLIT and NTRK-like protein 1	Slitrk1	77.8	2	1	48	Plasma membrane

*http://amigo.geneontology.org/amigo/landing

Supplementary Table 3

Supplementary results for mass-spectrometry analysis (supplementary results for Figure 9a). Arrow and arrowhead in Figure 9a correspond to the excised bands of 200 kDa and 100 kDa, respectively. Candidate proteins were identified by Proteome Discoverer v2.4 (Thermo Fisher Scientific) against the MASCOT algorithm with amino acid sequences in the SwissProt database.

Gene	Forward Primer	Reverse Primer
Sema3a	GAAGAGCCCTTATGATCCCAAAC	AGATAGCGAAGTCCCGTCCC
Sema3c	ATGGCATTCCGGGCGATTT	GGTTTTGGTTTCTCGAAGCTCA
Sema3f	TGCTACCCCTATCCAGGACC	CTGTAGTCTGTAGTGTTGAGCAG
Sema7a	ACACACCGTGCTTTTCCATGA	CCTTTGTGGAGCCGATGTTC
EphA5	ACACGTCCAAAGGGTTCAAGA	GTACGGTGTCATTTGTTGGTCT
EphB2	ATTATTTGCCCCAAAGTGGACTC	GCAGCGGGGTATTCTCCTTC
Slit1	CAAGAACATCCCACGGAACAC	TCCACAGCTCCGATCTGGTT
Slit2	CCATGTAAAAATGATGGCACCTG	ATCACAGTCCTGACCCTTGAA
Slit3	TGCCCCACCAAGTGTACCT	GGCCAGCGAAGTCCATTTTG
BMP2	GGGACCCGCTGTCTTCTAGT	TCAACTCAAATTCGCTGAGGAC
Netrin	CAGCCTGATCCTTGCTCGG	GCGGGTTATTGAGGTCGGTG
Wnt5a	CAACTGGCAGGACTTTCTCAA	CATCTCCGATGCCGGAACT
Wnt7b	TTTGGCGTCCTCTACGTGAAG	CCCCGATCACAATGATGGCA
Shh	AAAGCTGACCCCTTTAGCCTA	TTCGGAGTTTCTTGTGATCTTCC
Ntf3	GGAGTTTGCCGGAAGACTCTC	GGGTGCTCTGGTAATTTTCCTTA
BDNF	TCATACTTCGGTTGCATGAAGG	AGACCTCTCGAACCTGCCC
GDNF	TCCAACTGGGGGTCTACGG	GCCACGACATCCCATAACTTCAT
MAO_A	GCCCAGTATCACAGGCCAC	CGGGCTTCCAGAACCAAGA
MAO_B	ATGAGCAACAAAAGCGATGTGA	TCCTAATTGTGTAAGTCCTGCCT
COMT	CACAGTGAAACTCAAAGTTACAGACA	GCAACAGGAGACCCAATGA
Alpha2a	TAGAACTGACTTTTCTTCCGTTCTC	AACATACACGCTCTTCTTCAAGC
VMAT2	CAACTTTGGAGTTGGTTTTGC	CCACCAGGTAGCCCATGATA
Slitrk1	GAAGGGGACTTACACGTAGACT	AGTGAGGGAATTGCCATGCAG

Supplementary Table 4
List of primers for quantitative PCR analysis.
