# Supplementary material for: SLITRK1-mediated noradrenergic projection suppression in the neonatal prefrontal cortex
Source: Commun Biol. 2022 Sep 9;5:935. doi: 10.1038/s42003-022-03891-y (PMC9463131; doi:10.1038/s42003-022-03891-y)
Supplement: Supplementary file 3 — Description of Additional Supplementary Data [file 42003_2022_3891_MOESM3_ESM.docx]

**Description of Additional Supplementary Files**

**File name:** Supplementary Data 1

**Description:** The source data behind the graphs in the paper
