# Supplementary material for: SLITRK1-mediated noradrenergic projection suppression in the neonatal prefrontal cortex
Source: Commun Biol. 2022 Sep 9;5:935. doi: 10.1038/s42003-022-03891-y (PMC9463131; doi:10.1038/s42003-022-03891-y)
Supplement: Supplementary file 5 — Reporting summary [file 42003_2022_3891_MOESM5_ESM.pdf]

nature portfolio

Corresponding author(s): Jun Aruga

Last updated by author(s): Aug 10, 2022

Reporting Summary

Nature Portfolio wishes to improve the reproducibility of the work that we publish. This form provides structure for consistency and transparency in reporting. For further information on Nature Portfolio policies, see our Editorial Policies and the Editorial Policy Checklist.

Statistics

For all statistical analyses, confirm that the following items are present in the figure legend, table legend, main text, or Methods section.

n/a	Confirmed	
☐	☒	The exact sample size (*n*) for each experimental group/condition, given as a discrete number and unit of measurement
☐	☒	A statement on whether measurements were taken from distinct samples or whether the same sample was measured repeatedly
☐	☒	The statistical test(s) used AND whether they are one- or two-sided<br>*Only common tests should be described solely by name; describe more complex techniques in the Methods section.*
☐	☒	A description of all covariates tested
☐	☒	A description of any assumptions or corrections, such as tests of normality and adjustment for multiple comparisons
☐	☒	A full description of the statistical parameters including central tendency (e.g. means) or other basic estimates (e.g. regression coefficient) AND variation (e.g. standard deviation) or associated estimates of uncertainty (e.g. confidence intervals)
☐	☒	For null hypothesis testing, the test statistic (e.g. *F*, *t*, *r*) with confidence intervals, effect sizes, degrees of freedom and *P* value noted<br>*Give P values as exact values whenever suitable.*
☒	☐	For Bayesian analysis, information on the choice of priors and Markov chain Monte Carlo settings
☒	☐	For hierarchical and complex designs, identification of the appropriate level for tests and full reporting of outcomes
☐	☒	Estimates of effect sizes (e.g. Cohen's *d*, Pearson's *r*), indicating how they were calculated

Our web collection on statistics for biologists contains articles on many of the points above.

Software and code

Policy information about availability of computer code

Data collection	Microscopic images, ZEN (Zeiss), Axiovision (Zeiss), Fluoview (Olympus)
Data analysis	Statistics, Microsoft Excel (Microsoft), SPSS statistical package (ver. 16, SPSS Inc.), BellCurve for Excel (Social Survey Research Information); Ultrasonic vocalization, SASLab Pro Recorder Software (Avisoft Bioacoustics); open field test, Image J OF4 (O'hara); morphometric analysis for immunostaining images, ImageJ (https://imagej.nih.gov/ij/), Neurolucida (MBF Bioscience); monoamine content, PowerChrom (EPC-500, Eicom); mass spectrometry, Proteome Discoverer v2.4 (Thermo Fisher Scientific).

For manuscripts utilizing custom algorithms or software that are central to the research but not yet described in published literature, software must be made available to editors and reviewers. We strongly encourage code deposition in a community repository (e.g. GitHub). See the Nature Portfolio guidelines for submitting code & software for further information.

Data

Policy information about availability of data

All manuscripts must include a data availability statement. This statement should provide the following information, where applicable:

- Accession codes, unique identifiers, or web links for publicly available datasets
- A description of any restrictions on data availability
- For clinical datasets or third party data, please ensure that the statement adheres to our policy

All data generated during this study are included in this published article and its supplementary information files.

Human research participants

Policy information about studies involving human research participants and Sex and Gender in Research.

Reporting on sex and gender	Control, male 454, female 606;<br>Schizophrenia, male 515, female 525;<br>Bipolar disorder, male 180, female 184.
Population characteristics	All the subjects were recruited from the Honshu area of Japan (the main island of Japan), where the population fall into a single genetic cluster (Yamaguchi-Kabata et al., Am. J. Hum. Genet. 83 (4), 445–456, 2008). Using a subset of subjects we previously showed that population stratification is negligible in our samples (Hattori et al., Am. J. Med. Genet. B Neuropsychiatr. Genet. 150 (8), 1110–1117, 2009; Yamada et al., PLoS One 6 (6), e20468, 2011). Best-estimate lifetime diagnosis of patients was made by direct interview with at least two experienced psychiatrists, according on DSM-IV criteria, and using all available information from medical records, hospital staff, and family informants.
Recruitment	Patients were recruited by multiple research institutes/hospitals in Japan. Control subjects were recruited from hospital staff and company employees documented to be free from psychoses and were further interviewed by experienced psychiatrists to exclude any psychiatric disorders.
Ethics oversight	The study was approved by the ethics committees of RIKEN and all participating institutes, and was conducted in accordance with the Declaration of Helsinki.

Note that full information on the approval of the study protocol must also be provided in the manuscript.

Field-specific reporting

Please select the one below that is the best fit for your research. If you are not sure, read the appropriate sections before making your selection.

☐ Life sciences ☐ Behavioural & social sciences ☐ Ecological, evolutionary & environmental sciences

For a reference copy of the document with all sections, see nature.com/documents/nr-reporting-summary-flat.pdf

Life sciences study design

All studies must disclose on these points even when the disclosure is negative.

Sample size	The sample sizes for each experiment were determined such that the power and significance in the two-sided test were 80 and 5%, respectively, according to Festing (On determining sample size in experiments involving laboratory animals. Lab. Anim. 52, 341350. 2018. doi: 10.1177/0023677217738268). However, the number of samples from the animals was minimized empirically.
Data exclusions	Outliers were removed after Grubbs' test for monoamine content analysis (Figure 4) and total branch length analysis in early neurite development assay (Figure 8c).
Replication	All attempts at replication were successful.
Randomization	All data were collected in sample-order-randomized manners.
Blinding	The experiments for assessing the new SLITRK1 mutations were carried out in a plasmid identity-blinded manner. Experimenters who were blinded to the specimen identity performed neurite analyses. Behavioral test was carried out in animal identity-blinded manner.

Behavioural & social sciences study design

All studies must disclose on these points even when the disclosure is negative.

Study description	*Briefly describe the study type including whether data are quantitative, qualitative, or mixed-methods (e.g. qualitative cross-sectional, quantitative experimental, mixed-methods case study).*
Research sample	*State the research sample (e.g. Harvard university undergraduates, villagers in rural India) and provide relevant demographic information (e.g. age, sex) and indicate whether the sample is representative. Provide a rationale for the study sample chosen. For studies involving existing datasets, please describe the dataset and source.*
Sampling strategy	*Describe the sampling procedure (e.g. random, snowball, stratified, convenience). Describe the statistical methods that were used to predetermine sample size OR if no sample-size calculation was performed, describe how sample sizes were chosen and provide a*

	rationale for why these sample sizes are sufficient. For qualitative data, please indicate whether data saturation was considered, and what criteria were used to decide that no further sampling was needed.
Data collection	*Provide details about the data collection procedure, including the instruments or devices used to record the data (e.g. pen and paper, computer, eye tracker, video or audio equipment) whether anyone was present besides the participant(s) and the researcher, and whether the researcher was blind to experimental condition and/or the study hypothesis during data collection.*
Timing	*Indicate the start and stop dates of data collection. If there is a gap between collection periods, state the dates for each sample cohort.*
Data exclusions	*If no data were excluded from the analyses, state so OR if data were excluded, provide the exact number of exclusions and the rationale behind them, indicating whether exclusion criteria were pre-established.*
Non-participation	*State how many participants dropped out/declined participation and the reason(s) given OR provide response rate OR state that no participants dropped out/declined participation.*
Randomization	*If participants were not allocated into experimental groups, state so OR describe how participants were allocated to groups, and if allocation was not random, describe how covariates were controlled.*

Ecological, evolutionary & environmental sciences study design

All studies must disclose on these points even when the disclosure is negative.

Study description	*Briefly describe the study. For quantitative data include treatment factors and interactions, design structure (e.g. factorial, nested, hierarchical), nature and number of experimental units and replicates.*
Research sample	*Describe the research sample (e.g. a group of tagged Passer domesticus, all Stenocereus thurberi within Organ Pipe Cactus National Monument), and provide a rationale for the sample choice. When relevant, describe the organism taxa, source, sex, age range and any manipulations. State what population the sample is meant to represent when applicable. For studies involving existing datasets, describe the data and its source.*
Sampling strategy	*Note the sampling procedure. Describe the statistical methods that were used to predetermine sample size OR if no sample-size calculation was performed, describe how sample sizes were chosen and provide a rationale for why these sample sizes are sufficient.*
Data collection	*Describe the data collection procedure, including who recorded the data and how.*
Timing and spatial scale	*Indicate the start and stop dates of data collection, noting the frequency and periodicity of sampling and providing a rationale for these choices. If there is a gap between collection periods, state the dates for each sample cohort. Specify the spatial scale from which the data are taken*
Data exclusions	*If no data were excluded from the analyses, state so OR if data were excluded, describe the exclusions and the rationale behind them, indicating whether exclusion criteria were pre-established.*
Reproducibility	*Describe the measures taken to verify the reproducibility of experimental findings. For each experiment, note whether any attempts to repeat the experiment failed OR state that all attempts to repeat the experiment were successful.*
Randomization	*Describe how samples/organisms/participants were allocated into groups. If allocation was not random, describe how covariates were controlled. If this is not relevant to your study, explain why.*
Blinding	*Describe the extent of blinding used during data acquisition and analysis. If blinding was not possible, describe why OR explain why blinding was not relevant to your study.*

Did the study involve field work? ☐ Yes ☐ No

Field work, collection and transport

Field conditions	*Describe the study conditions for field work, providing relevant parameters (e.g. temperature, rainfall).*
Location	*State the location of the sampling or experiment, providing relevant parameters (e.g. latitude and longitude, elevation, water depth).*
Access & import/export	*Describe the efforts you have made to access habitats and to collect and import/export your samples in a responsible manner and in compliance with local, national and international laws, noting any permits that were obtained (give the name of the issuing authority, the date of issue, and any identifying information).*
Disturbance	*Describe any disturbance caused by the study and how it was minimized.*

Reporting for specific materials, systems and methods

We require information from authors about some types of materials, experimental systems and methods used in many studies. Here, indicate whether each material, system or method listed is relevant to your study. If you are not sure if a list item applies to your research, read the appropriate section before selecting a response.

Materials & experimental systems

n/a	Involved in the study
☐	☒ Antibodies
☐	☒ Eukaryotic cell lines
☒	☐ Palaeontology and archaeology
☐	☒ Animals and other organisms
☒	☐ Clinical data
☒	☐ Dual use research of concern

Methods

n/a	Involved in the study
☒	☐ ChIP-seq
☒	☐ Flow cytometry
☒	☐ MRI-based neuroimaging

Antibodies

Antibodies used

anti-SLITRK1 rabbit antibody, anti SLITRK1 ECD antibody (AF3009, R&D, 1:5000), anti-tyrosine hydroxylase (#AB152, Millipore, 1:1000), anti-NET (NET05-01, Mab technologies, Inc., 1:5000), anti-ChAT (AB144P, Millipore, 1:300), anti-SERT (HTT-Rb-Af560, Frontier Institute, 1:300), anti-DAT (MAB369, Millipore, 1:1000), anti-green fluorescent protein (GFP) (#598, MBL, 1:1000), anti-VGLUT1 (NeuroMab, N28/9, 1:500), anti-VGAT (#131 003, Synaptic Systems, 1:1000), anti-microtubule-associated protein 2 (MAP2) (AB5622, Millipore, 1:1000), anti-HA (3F10, Sigma, 1:1000), and anti-phospho-extracellular signal-regulated kinases (SC-7383, Santa Cruz, 1:200), anti-L1CAM (MAB5272, Chemicon, 1:1,000), anti-Neurofascin (A12/18, NeuroMab, 1:10,000), and anti-NCAM (MAB310, Chemicon, 1:1,000), anti Dynamin1 (MAB5402, Chemicon, 1:5000), anti-His tag (H-15, Santa Cruz, 1:5000), anti-HA rabbit (H6908, Sigma-Aldrich, 1:1000), anti-actin (A2066, Sigma-Aldrich, 1:5000).

Validation

Primary antibody anti-SLITRK1 rabbit antibody was previously validated (Ref 27, Mol. Psychiatry 15, 177-184 (2010)). The other primary antibodies were validated by manufacturers as follows:
anti-tyrosine hydroxylase (#AB152, Millipore), https://www.merckmillipore.com/JP/ja/product/Anti-Tyrosine-Hydroxylase-Antibody,MM_NF-AB152
anti-green fluorescent protein (#598, MBL), https://ruo.mbl.co.jp/bio/dtl/A/?pcd=598
anti-VGLUT1 (N28/9, NeuroMab), https://www.antibodiesinc.com/products/anti-vglut1-antibody-n28-9-75-066
anti-VGAT (#131 003, Synaptic Systems), https://sysy.com/product/131003
anti-microtubule-associated protein 2 (AB5622, Millipore), https://www.merckmillipore.com/JP/ja/product/Anti-Microtubule-Associated-Protein-2-MAP2-Antibody,MM_NF-AB5622
anti-HA (3F10, Sigma-Aldrich), https://www.sigmaaldrich.com/JP/ja/product/roche/roahaha
anti actin antibody (A2066, Sigma-Aldrich), https://www.sigmaaldrich.com/JP/ja/product/sigma/a2066
anti-ChAT (AB144P, Millipore), https://www.sigmaaldrich.com/JP/ja/product/mm/ab144p
anti-DAT (MAB369, Millipore), https://www.merckmillipore.com/JP/ja/product/Anti-Dopamine-Transporter-Antibody-NT-clone-DAT-Nt,MM_NF-MAB369
anti SLITRK1 ECD antibody (AF3009, R&D), https://www.rndsystems.com/products/human-slitrk1-antibody_af3009
anti-NET (NET05, Mab technologies), https://mabtechnologies.com/categories/product/6-norepinephrine-transporter-mouse-net05-1
anti NCAM (MAB310, Chemicon), https://www.labome.com/knockout-validated-antibodies/NCAM-antibody-knockout-validation-Millipore-AB5032.html
anti Neurofascin (A12/18, NeuroMab), https://www.antibodiesinc.com/products/anti-pan-neurofascin-extracellular-antibody-a12-18-75-172
anti His tag antibody (H-15, Santa Cruz), https://www.scbt.com/ja/p/his-probe-antibody-h-15
Anti HA rabbit antibody (H6908, Sigma-Aldrich), https://www.sigmaaldrich.com/JP/ja/product/sigma/h6908
anti L1CAM (MAB5272, Chemicon), https://www.sigmaaldrich.com/JP/ja/product/mm/mab5272?gclid=CjwKCAjwtIaVBhBkEiwAsr7-cwgxTFzrLgSV9q3BTcaFfaiciTYIdYTpbHjqZ1fb7UpJSbuXn5WLpxoCLRQQAvD_BwE
anti Dynamin1 antibody (MAB5402, Chemicon), https://www.sigmaaldrich.com/JP/ja/product/mm/mab5402
anti-SERT (HTT-Rb-Af560, Frontier Institute), https://nittobo-nmd.co.jp/pdf/reagents/HTT.pdf

Eukaryotic cell lines

Policy information about cell lines and Sex and Gender in Research

Cell line source(s)

COS7 cells, HEK293T cells, and PC12 cells were obtained from RIKEN Bioresource Center Cell Bank. PC12D cells were provided by Dr. Shinichi Hisanaga (Tokyo Metropolitan University), and were originally developed in Katoh-Semba, R., Kitajima, S., Yamazaki, Y. & Sano, M. Neuritic growth from a new subline of PC12 pheochromocytoma cells: Cyclic AMP mimics the action of nerve growth factor. J. Neurosci. Res. 17, 36-44 (1987).

Authentication

None of the cell lines used were authenticated.

Mycoplasma contamination

The cell lines were not tested for mycoplasma contamination.

Commonly misidentified lines (See ICLAC register)	No commonly misidentified cell lines were used in this study.

Animals and other research organisms

Policy information about studies involving animals; ARRIVE guidelines recommended for reporting animal research, and Sex and Gender in Research

Laboratory animals	C57BL/6J mice, CD-1 mice were purchased from Japan SLC and CLEA Japan, Slitrk1-deficient mice (ref 27, http://www.informatics.jax.org/allele/MGI:3834477) were maintained in RIKEN Brain Science Institute and Nagasaki University. All animal experiments were conducted following the guidelines for animal experimentation in RIKEN and Nagasaki University.
Wild animals	Wild animals were not used.
Reporting on sex	The sex of the used animals are indicated in all data.
Field-collected samples	Field collected samples were not used.
Ethics oversight	All animal experiments were approved by Animal Experiment Committees at the RIKEN Brain Science Institute and Animal Care and Use Committee of Nagasaki University.

Note that full information on the approval of the study protocol must also be provided in the manuscript.
